# Concordance in detecting amyloid positivity between ^18^F-florbetaben and ^18^F-flutemetamol amyloid PET using quantitative and qualitative assessments

**DOI:** 10.1038/s41598-020-76102-5

**Published:** 2020-11-11

**Authors:** Soo Hyun Cho, Yeong Sim Choe, Young Ju Kim, Byungju Lee, Hee Jin Kim, Hyemin Jang, Jun Pyo Kim, Young Hee Jung, Soo-Jong Kim, Byeong C. Kim, Gill Farrar, Duk L. Na, Seung Hwan Moon, Sang Won Seo

**Affiliations:** 1 Department of Neurology, Samsung Medical Center, Sungkyunkwan University School of Medicine, Seoul, Republic of Korea; 2 Department of Neurology, Chonnam National University Medical School, Chonnam National University Hospital, Gwangju, Korea; 3 Department of Health Sciences and Technology, SAIHST, Sungkyunkwan University, Seoul, Korea; 4 Neuroscience Center, Samsung Medical Center, Seoul, Korea; 5 Department of Neurology, Yuseong Geriatric Rehabilitation Hospital, Pohang, Korea; 6 Department of Neurology, Myoungji Hospital, Hanyang University, Goyangsi, Korea; 7 Pharmaceutical Diagnostics, GE Healthcare, Chalfont St Giles, UK; 8 Stem Cell and Regenerative Medicine Institute, Samsung Medical Center, Seoul, Korea; 9 Department of Nuclear Medicine, Sungkyunkwan University School of Medicine, Samsung Medical Center, 81 Irwon-ro, Gangnam-gu, Seoul, 06351 Republic of Korea; 10 Samsung Alzheimer Research Center, Samsung Medical Center, Seoul, Korea; 11 Department of Intelligent Precision Healthcare Convergence, Sungkyunkwan University School of Medicine, Suwon, Korea

**Keywords:** Biomarkers, Neurology

## Abstract

We aimed to quantitatively and qualitatively assess whether there is a discrepancy in detecting amyloid beta (Aβ) positivity between 18F-florbetaben (FBB) and 18F-flutemetamol (FMM) positron emission tomography (PET). We obtained paired FBB and FMM PET images from 107 participants. Three experts visually quantified the Aβ deposition as positive or negative. Quantitative assessment was performed using global cortical standardized uptake value ratio (SUVR) with the whole cerebellum as the reference region. Inter-rater agreement was excellent for FBB and FMM. The concordance rates between FBB and FMM were 94.4% (101/107) for visual assessment and 98.1% (105/107) for SUVR cut-off categorization. Both FBB and FMM showed high agreement rates between visual assessment and SUVR positive or negative categorization (93.5% in FBB and 91.2% in FMM). When the two ligands were compared based on SUVR cut-off categorization as standard of truth, although not statistically significant, the false-positive rate was higher in FMM (9.1%) than in FBB (1.8%) (*p* = 0.13). Our findings suggested that both FBB and FMM had excellent agreement when used to quantitatively and qualitatively evaluate Aβ deposits, thus, combining amyloid PET data associated with the use of different ligands from multi-centers is feasible.

## Introduction

Amyloid positron emission tomography (PET) is a widely used biomarker-supported method for diagnosing Alzheimer’s disease (AD)^1^. To determine amyloid beta (Aβ) peptide deposition positivity, visual assessment is generally performed by an expert and quantitative assessment is used for research purposes^2^. Visual assessment is determined primarily by tracer uptake in grey matter. Previously, visual assessment showed high agreement with autopsy findings, however, the results may differ depending on inter-rater discrepancy, expert skill, and ligand type^3^.

A quantitative method for assessing amyloid deposition uses the cortical-to-reference region standardized uptake value ratio (SUVR). Although the SUVR method is objective and simple, there are several limitations including partial volume correction, image reconstruction and processing, region-of-interest (ROI) delineation method, definition of the standard of truth (SoT), and select of an appropriate reference region^4^.

F-labeled ligands are amyloid PET ligands widely used for diagnosing AD. Among them, ^18^F-florbetaben (FBB)^5^ and ^18^F-flutemetamol (FMM)^6^ are widely used in Europe and Asia. FBB is an ^18^F-labeled polyethylene glycol stilbene derivative with high in vitro affinity and specificity for Aβ plaques^7^. FMM is the ^18^F-labelled analogue of ^11^C-PiB and shows strong concordance with histopathology for brain fibrillar Aβ. FMM has been useful for differentiating cognitively normal people from AD patients with high specificity and sensitivity for detection of AD^8^. Although scanning protocols are relatively similar across the tracers, FDA-approved visual rating guidelines to determine a scan positive or negative differ considerably. These differences include color scale used, intensity scaling, region definitions, as well as spatial and signal thresholds to determine positivity. When amyloid PET is taken using different ligands in the same person, reading results are sometimes different.

In the present study, the discrepancy in detecting amyloid positivity between FBB and FMM PET was investigated using visual assessment, SUVR and direct comparison of FBB-FMM Centiloid (dcCL) cut-off categorization. In addition, the discrepancy rate between visual assessment and SUVR cut-off categorization in FBB and FMM was examined. Ideally, histopathological confirmation of Aβ presence in the brain should be the SoT. However, this analysis is rarely achievable because it must be performed post-mortem. Therefore, the false-positive and false-negative rates were compared between the two ligands based on visual assessment and SUVR cut-off categorization as SoT.

## Results

### Participant demographics

Table 1 presents participant demographic information. The average age (mean ± standard deviation (SD)) of all 107 participants was 64.4 ± 17.2 years and 56.1% were females. Frequency of apolipoprotein E (APOE) ε4 status was 58.5% in non-carriers, 31.1% in heterozygous, and 10.4% in homozygous participants. The Mini-Mental State Examination (MMSE) score for all participants was 26.2 ± 5.0.

**Table 1:** Participant demographics and clinical findings.

Characteristics	
Number of participants (Number (%))	YC/OC/MCI/ADD/SVAD 20 (18.7)/27 (25.2)/27 (25.2)/29 (27.1)/4 (3.7)
Age (mean ± SD)	64.4 ± 17.2
Sex (female no. (%))	60 (56.1)
APOE ε4, no. (%) (0/1/2)	62 (58.5)/33 (31.1)/11 (10.4)
MMSE (mean ± SD)	26.2 ± 5.0

Statistical analyses were performed using chi-square tests for sex and APOE ε4 and ANOVA for age and MMSE.

*YC* young control, *CN* cognitively normal, *OC* old control, *MCI* mild cognitive impairment, *ADD* Alzheimer’s disease dementia, *SVAD* subcortical vascular dementia, *MMSE* Mini-Mental State Examination, *APOE ε4* apolipoprotein E ε4 allele, *ANOVA* analysis of variance, *SD* standard deviation.

### Concordance rate for visual assessment between FBB and FMM ligands

**Figure 1:**
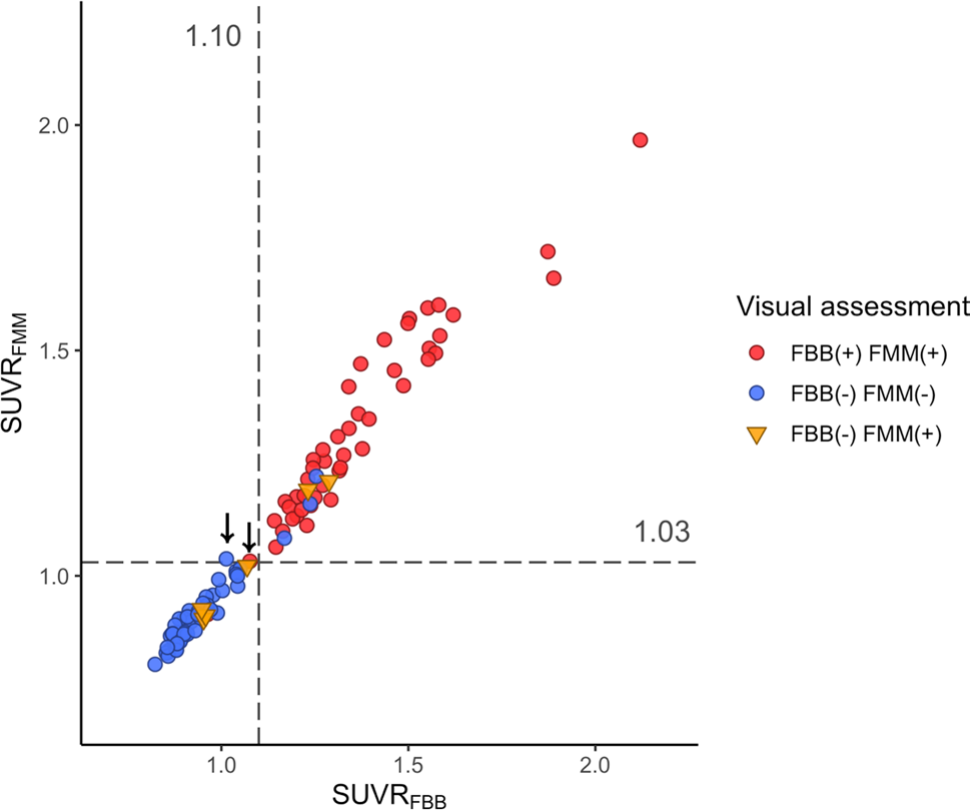
Scatter plot of FBB and FMM SUVR with WC as the reference region. The scatter plot is the result of visual assessment based on SUVR values. The cortical SUVR cut-off value was 1.10 for FBB and 1.03 for FMM when WC was used as the reference region. Two discordant SUVR positivity participants are represented with an arrow and their SUVR positivity was both FBB-negative and FMM-positive. The six participants whose visual assessment results were discordant between FBB and FMM are represented with an inverted triangle. Abbreviations: FBB, ^18^F-florbetaben; FMM, ^18^F-flutemeta.mol; SUVR, standardized uptake value ratio; WC, whole cerebellum.

**Figure 2:**
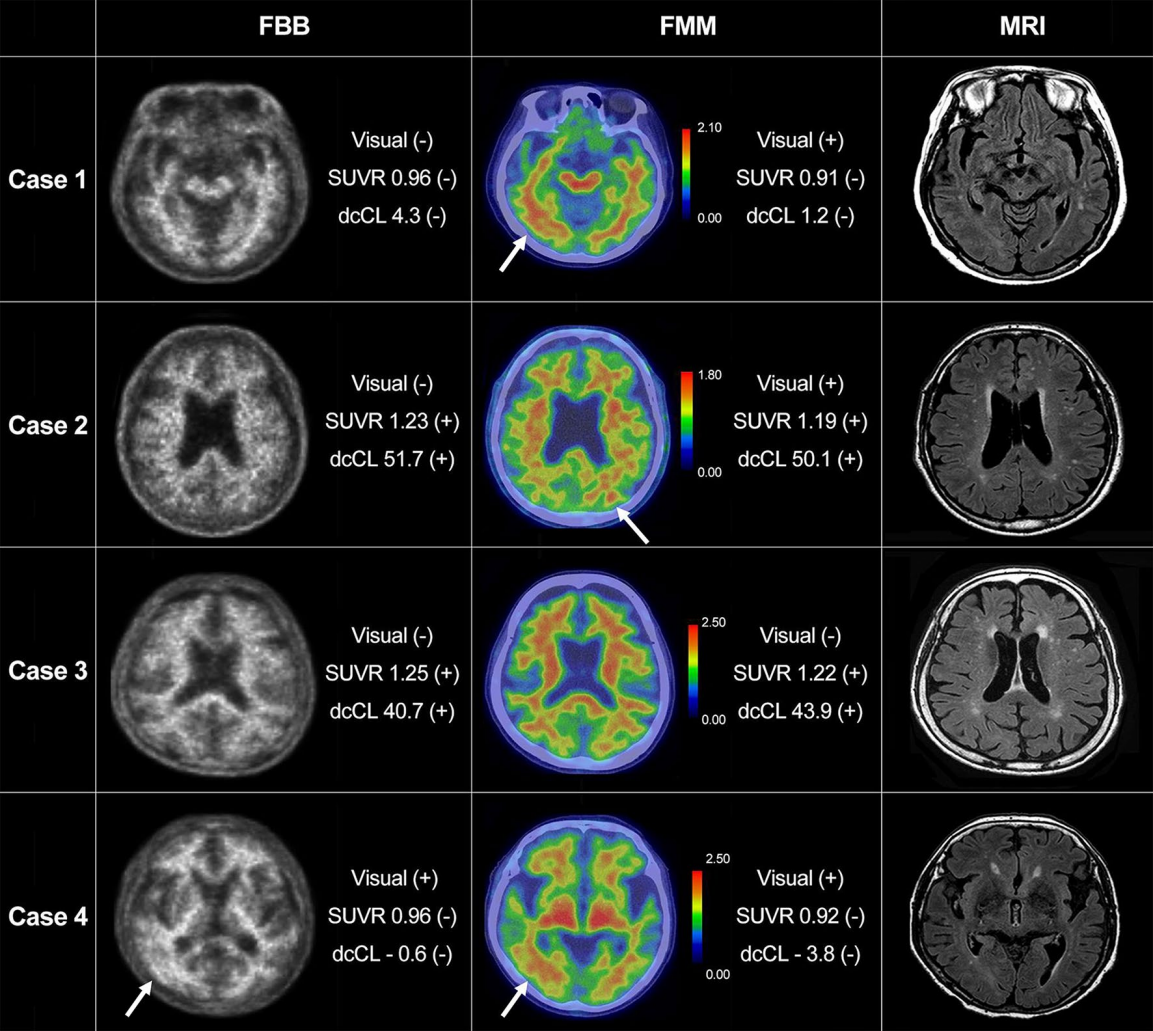
FBB and FMM uptake in participants. Four representative cases of FBB and FMM PET and FLAIR MRI are shown. Case 1 represents the participant who was FBB-negative but FMM-positive based on visual assessment although SUVR and dcCL positivity were both negative. Case 2 represents the participant who was FBB-negative but FMM-positive based on visual assessment although SUVR and dcCL positivity were both positive. Case 3 represents the participant whose visual assessment was negative but SUVR and dcCL positivity were both positive for FBB and FMM. Case 4 represents the participant who was positive based on visual assessment but SUVR and dcCL positivity were both negative for FBB and FMM. The Scale bar indicates standardized uptake values (SUVs). The arrow indicates focal uptake of FMM in the cortex. Abbreviations: FBB, ^18^F-florbetaben, FMM, ^18^F-flutemetamol; FLAIR, fluid-attenuated inversion recovery; SUVR, standardized uptake value ratio; dcCL, the Centiloid (CL) units using the direct comparison of FBB-FMM CL method.

The concordance rate for visual assessment between FBB and FMM was 94.4% (101/107, Fig. 1 and Table 2). In six participants with discordant results, all were FBB-negative and FMM-positive. In Fig. 2, case 1 represents the participant who was FBB-negative but FMM-positive based on visual assessment although SUVR and dcCL positivity were both negative. Case 2 represents the participant who was FBB-negative but FMM-positive based on visual assessment although SUVR and dcCL positivity were both positive.

**Table 2:** Characteristics of six visual assessment-discordant participants between FBB and FMM.

	Diagnosis	Age	Sex	FBB visual assessment	FMM visual assessment	FBB SUVR	FMM SUVR	FBB_dcCL	FMM_dcCL
1	CN	77	M	Negative	Positive	0.95 (N)	0.9 (N)	6.2 (N)	3.2 (N)
2	MCI	74	F	Negative	Positive	0.96 (N)	0.91 (N)	4.3 (N)	1.2 (N)
3	MCI	64	M	Negative	Positive	0.95 (N)	0.93 (N)	7.0 (N)	6.0 (N)
4	MCI	77	F	Negative	Positive	1.07 (N)	1.02 (N)	37.6 (P)	34.6 (P)
5	MCI	79	M	Negative	Positive	1.23 (P)	1.19 (P)	51.7 (P)	50.1 (P)
6	ADD	69	F	Negative	Positive	1.29 (P)	1.21 (P)	66.1 (P)	57.8 (P)

The visual assessment results with different positivity between FBB and FMM and their SUVR. The cortical SUVR cut-off value was 1.1 for FBB and 1.03 for FMM when WC was used as the reference region. The dcCL cut-off value was 24.9 dcCL units for FBB and 15.1 dcCL units for FMM when WC was used as the reference region.

*FBB* ^18^F-florbetaben, *FMM* ^18^F-flutemetamol, *SUVR* standardized uptake value ratio, *dcCL* the Centiloid (CL) units using the direct comparison of FBB-FMM CL method, *CN* cognitively normal, *MCI* mild cognitive impairment, *ADD* Alzheimer’s disease dementia, *N* Negative, *P* Positive.

### Concordance rate for SUVR and dcCL cut-off categorization between FBB and FMM ligands

When comparing SUVR positivity between FBB and FMM, a high concordance rate was achieved (105/107 = 98.1%). Two patients with discordant SUVR positivity were FBB-negative and FMM-positive (Fig. 1 and Table 3); their SUVRs were near the cut-off value (1.01 and 1.08 for FBB SUVR cut-off [1.10] and 1.04 and 1.03 for FMM SUVR cut-off values [1.03]). Visual assessment of the two participants showed the same results: one was positive and the other was negative for both FBB and FMM.

**Table 3:** Characteristics of participants with discordant results between visual assessment and SUVR assessment on either FBB or FMM PET.

FBB/FMM SUVR	Sex	Age	Group	FBB visual	FMM visual	FBB SUVR	FMM SUVR	AD-specific pattern similarity^a^
Negative/negative	M	80	ADD	Positive	Positive	0.96	0.91	98.90
	M	77	CN	Negative	Positive	0.95	0.90	18.57
	F	74	MCI	Negative	Positive	0.96	0.91	24.83
	M	64	MCI	Negative	Positive	0.95	0.93	9.43
	F	77	MCI	Negative	Positive	1.07	1.02	98.90
Negative/positive	M	66	MCI	Negative	Negative	1.01	1.04	39.21
	F	75	ADD	Positive	Positive	1.08	1.03	50.65
Positive/positive	F	69	ADD	Negative	Positive	1.29	1.21	89.76
	F	77	MCI	Negative	Negative	1.17	1.08	53.93
	F	84	MCI	Negative	Negative	1.24	1.16	93.38
	F	83	ADD	Negative	Negative	1.25	1.22	95.34
	M	79	MCI	Negative	Positive	1.23	1.19	14.30

*FBB* ^18^F-florbetaben, *FMM* ^18^F-flutemetamol, *SUVR* standardized uptake value ratio, *CN* cognitively normal, *MCI* mild cognitive impairment, *ADD* Alzheimer’s disease dementia.

^a^In previous research^9^, we analysed the cortical atrophy pattern for each subject based on the cortical thickness data and measure the AD-specific pattern similarity then calculated on an individual subject basis.

**Figure 3:**
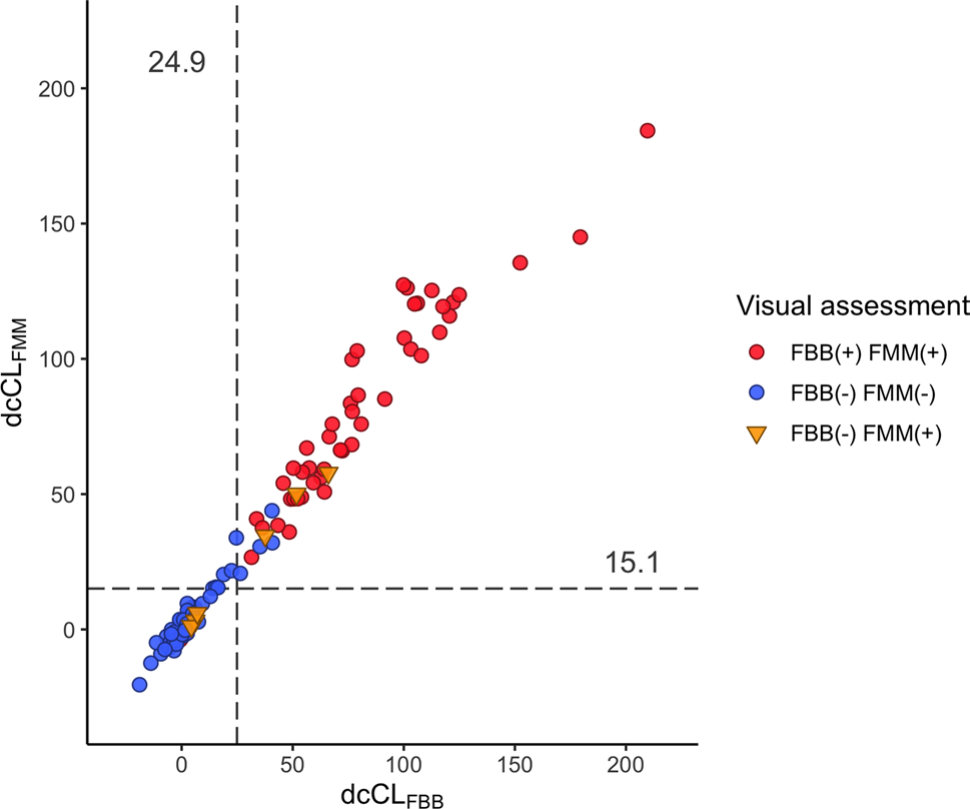
Scatter plot of FBB and FMM dcCL values with WC as the reference region. The scatter plot is the result of visual assessment based on the dcCL values. The dcCL cut-off value was 24.9 dcCL units for FBB and 15.1 dcCL units for FMM when WC was used as the reference region. There were six discordant patients whose dcCL positivity was FBB-negative and FMM-positive. The participants whose visual assessment results were discordant between FBB and FMM are represented with an inverted triangle. Abbreviations: FBB, ^18^F-florbetaben; FMM, ^18^F-flutemeta.mol; dcCL, the Centiloid (CL) units using the direct comparison of FBB-FMM CL method.

When converting SUVRs of FBB and FMM to dcCL, the concordance rate between FBB dcCL and FMM dcCL positivity was 94.4% (101/107; Fig. 3). There were six discordant patients whose dcCL positivity was FBB-negative and FMM-positive. Compared with the six visual discordant participants (FBB-negative and FMM-positive), the dcCL positivity for the six visual discordant participants showed three participants were positive and three participants were negative for both FBB and FMM (Table 2).

### Concordance rate between SUVR cut-off categorization and visual assessment for FBB and FMM ligands

For FBB, visual assessment and SUVR classification did not match in seven of 107 participants (Fig. 1): five participants were visually negative but SUVR-positive, and two participants were visually positive but SUVR-negative. For FMM, disagreement between visual and SUVR classification was found in nine of 107 participants: four participants were visually negative but SUVR-positive, and five participants were visually positive but SUVR-negative. In Fig. 2, case 3 represents the participant in which the visual assessment was negative but the SUVR classification positive for both FBB and FMM. In Fig. 2, case 4 shows the participant for whom the visual assessment was positive but the SUVR classification was negative for both FBB and FMM.

### False-positive and false-negative rates for FBB and FMM

**Figure 4:**
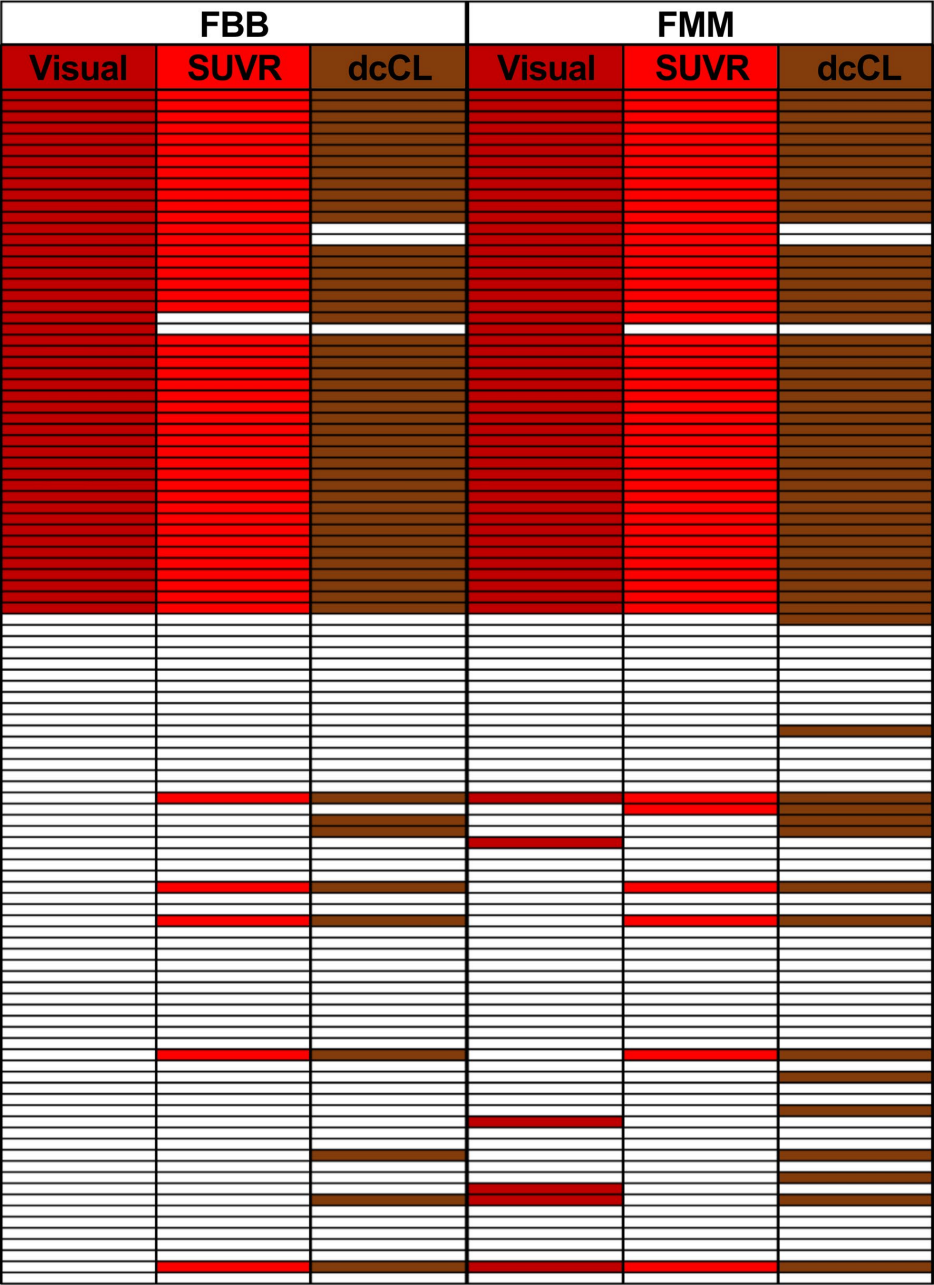
Positivity agreement among visual assessment, SUVR, and dcCL positivity in FBB and FMM. The visual, SUVR, and dcCL positivity results of FBB and FMM are shown as positive in red and negative in grey. Abbreviations: FBB, ^18^F-florbetaben, FMM, ^18^F-flutemetamol; SUVR, standardized uptake value ratio; dcCL, the Centiloid (CL) units using the direct comparison of FBB-FMM CL method.

Visual assessment was set as SoT among the 101 participants except for six participants whose visual assessment result did not match between FBB and FMM. For FBB, there were two false-negative (SUVR-negative but visual assessment-positive) and three false-positive (SUVR positive but visual assessment-negative) participants. For FMM, there was one false-negative participant (SUVR-negative but visual assessment-positive) and four false-positive (SUVR-positive but visual assessment-negative) participants (Fig. 4). The false-positive rate was 5.6% (3/54) for FBB and 7.4% (4/54) for FMM. The false-negative rate was 4.3% (2/47) for FBB and 2.1% (1/47) for FMM. In this case, statistical differences in false-positive rate (5.6% *vs*. 7.4%, *p* = 1.0) and false-negative rate (4.3% *vs*. 2.1%, *p* = 1.0) were not observed between FBB and FMM.

The SUVR cut-off categorization was set as SoT among the 105 participants except for two participants whose SUVR result did not match between FBB and FMM. For FBB, there was one false-positive participant (SUVR-negative but visual assessment-positive) and five false-negative participants (SUVR-positive but visual assessment-negative). For FMM, five participants were false-positive (SUVR-negative but visual assessment-positive) and three participants were false-negative (SUVR-positive but visual assessment-negative; Fig. 4). The false-positive rate was 1.8% (1/55) for FBB and 9.1% (5/55) for FMM. The false-negative rate was 10% (5/50) for FBB and 6% (3/50) for FMM. In this case, differences between the two ligands in false-positive rate (1.8% vs. 9.1%, *p* = 0.13) and false-negative rate (10% vs. 6%, *p* = 0.5) were not observed.

Previously, we developed a new method for measuring Alzheimer’s disease (AD)-specific similarity of cortical atrophy patterns at the individual-level^9^. The AD-specific atrophy similarity score represents how similar the cortical atrophy pattern of an individual participant is to that of a representative AD patient. In Table 3, SUVR-positive but visual assessment-negative cases (mean ± SD, 69.34 ± 35.14) seemed to show higher AD-specific pattern similarity than SUVR-negative but visual assessment-positive cases (50.13 ± 44.86, *p* = 0.65), although it is not statistically significant. However, the number of participants is very small that it is difficult to determine statistically.

## Discussion

In terms of visual assessment and SUVR cut-off categorization, the concordance rate for Aβ positivity between FBB and FMM was investigated in 107 participants who underwent both FBB and FMM PET for Aβ deposits. High agreement rates were found between FBB and FMM in visual assessment (94.4%) and SUVR cut-off categorization (98.1%). In addition, both FBB and FMM showed high agreement rates between visual assessment and SUVR cut-off categorization (93.5% in FBB and 91.6% in FMM). Furthermore, visual assessment or SUVR cut-off categorization as SoT produced the same results for the two ligands; false-positive and false-negative rates were not different between the two ligands. Taken together, these findings indicate that both FBB and FMM had excellent agreement when used to quantitatively and qualitatively evaluate Aβ deposits, thus, combining amyloid PET data associated with the use of different ligands from multi-centers can be useful in future research.

In the present study, inter-rater agreement was high for FBB (Fleiss k = 0.86) and FMM (Fleiss k = 0.78). The results are consistent with previous studies in which the inter-reader agreement was high in FBB (k = 0.89–0.94)^7^ and FMM (Fleiss k = 0.63–0.83)^10^.

High agreement rates were found between FBB and FMM in visual assessment (94.4%) and SUVR cut-off categorization (98.1%). The concordance rates for Aβ positivity among ^18^F-ligands have not yet been extensively evaluated in direct comparison studies. However, the results of the present study were supported by our previous work showing the spatial distribution of increased FMM uptake was similar to FBB^11^. In addition, FMM and FBB were highly correlated (R^2^ = 0.97) and showed similar dynamic ranges (slope = 0.99). Notably, the SUVR cut-off values (1.1 for FBB and 1.03 for FMM) using Whole cerebellum (WC) as the reference region were different from SUVR cut-offs proposed in previous studies (0.96 for FBB^4^ and 1.23 for FMM^12^). The discrepancy might be explained by the differences in target cortical regions, tools used for analysis, and the method of developing SUVR cut-offs (iterative outlier method with old controls (OCs) in this study compared with the receiver operating characteristic method in previous studies). When using the SUVR cut-off categorization, only two cases of Aβ positivity mismatch were observed between the two ligands although SUVR cut-off categorization values were determined for different participants.

In addition, both FBB and FMM showed high concordance rates between visual assessment and SUVR cut-off categorization for Aβ deposits (93.5% in FBB and 91.6% in FMM). The findings were consistent with previous studies in which the accuracies of visual assessment and quantitative assessment in evaluating Aβ positivity were comparable^2,3^. FBB (91%–96%)^4^ and FMM (95.3%)^13^ showed high agreement between visual assessment and SUVR quantification. In a previous study, disagreement among cases was possibly explained by severe brain atrophy^13^, however, severe atrophy was not observed in the present study. Most of the discrepancies between visual assessment and SUVR results were due to amyloid focal uptake in both FBB and FMM as shown in Fig. 2 case 4.

In the present study, based on visual assessment or SUVR cut-off categorization as SoT, both FBB and FMM showed low false-positive and false-negative rates. When the two ligands were compared based on visual assessment results as SoT, although not statistically significant, false-positive rates (visual assessment-negative, SUVR-positive, 5.6% for FBB and 7.4% for FMM) were greater than the false-negative rates (visual assessment-positive, SUVR-negative, 4.3% for FBB and 2.1% for FMM). The SUVRs in all false-positive cases were near cut-off values. All false-positive FBB cases also overlapped with FMM, therefore, these false-positive cases are likely true-positive although the visual interpretation of FBB and FMM imaging showed high sensitivity and specificity in detecting Aβ plaques^14,15^. Due to improved sensitivity and specificity, quantitative assessment is considered more accurate than visual assessment^16^. Therefore, visually assessed false-positive cases should be followed up to determine whether they clinically progress or a follow-up Aβ PET becomes positive.

When the two ligands were compared based on SUVR cut-off categorization as SoT, although not statistically significant, the false-positive rate (visual assessment-positive, SUVR-negative) was higher in FMM (9.1%) than in FBB (1.8%). Because the FBB and FMM showed minimal difference in SUVR positivity, these tracers are almost identical for assessment of amyloid deposition using quantitative measures. However, in some cases, although not many, visual interpretations were different between the tracers, possibly because regional uptakes were considered in the visual interpretation. Conversely, the average value of volume-weighted SUVRs from cerebral cortical volumes of interest (VOI) that determined positivity in a quantitative assessment, might not robustly reflect significant regional Aβ plaque burden. In our experience, assessing uptakes in the lateral temporal cortex was often more problematic than in other regions under visual analysis, especially near the occipital lobe border. The area was large, and the uptake often looked different depending on image tilting. In cases of ambiguous or indeterminate scans, the reading can be altered if some regions are over- or under-evaluated.

Why some FMM images were over-estimated is not evident. However, unlike FBB, FMM was evaluated based on a color scale, which might have affected the reading. Apparently, experts conducting visual analysis on a color scale tend to over-estimate the reading. In Fig. 2 case 1, which presents FMM-positive based only on visual assessment, there was focal uptake toward the lateral temporal cortex, while there was a visually distinguishable difference in signal activity between grey and white matter in the same FBB area. In the present study, signal differences were better discriminated using FBB than FMM. In addition, FMM appears slightly blurrier than FBB, which might be another reason for the discrepancy. Alternatively, when pathology and visual reading in FMM were compared in previous studies, more false-positives were identified with visual reading, apparently due to the presence of diffuse plaques^14^. In addition, FMM binds to both neuritic and diffuse Aβ plaques^14,17^. Both types of plaque generally co-exist in the neocortex of AD patients and FMM PET signal corresponds predominantly to neuritic plaques but is affected by the presence of diffuse plaques^18^. The additional FMM PET signal from diffuse Aβ plaques can result in positive PET reads, which might contribute to the false positivity of FMM seen in this study in addition to the aforementioned factor.

The strength of the present study was the direct comparison of two different amyloid tracers in the same subjects using standardized protocols and the same type of scanners. However, the present study had several limitations. First, pathologic verification was lacking and pathologic Aβ burdens should be used in further studies to validate the results. Second, the generalization of the results might be difficult due to differences between PET scanners, acquisition protocols, and reconstruction methods at other sites. However, the findings provide valuable information on distinct features of FBB and FMM scans, which can be used for better evaluation of Aβ imaging across institutions and studies.

In conclusion, visual assessment and quantitative measurement of amyloid deposition were investigated based on direct comparison of FBB and FMM PET scans. Both FBB and FMM had excellent quantitative and qualitative agreement for evaluating Aβ deposits, thus, combining amyloid PET data associated with the use of different ligands from multi-centers is feasible.

## Methods

### Participants

In the present study, 20 young controls (YCs), 27 OCs, and 27 mild cognitive impairment (MCI), 29 AD dementia (ADD), and 4 subcortical vascular dementia (SVAD) patients were recruited. All participants underwent Aβ PET with both FBB and FMM as well as magnetic resonance imaging (MRI). ADD was diagnosed based on the National Institute on Aging-Alzheimer’s Association (NIA-AA) research criteria for probable ADD^19^. Participants diagnosed with MCI had to meet Petersen’s criteria^20^ and show objective memory impairment one SD below the norm in at least one memory test. The OCs were over 65 years of age with normal cognitive function determined using neuropsychological tests^21^ and no history of neurological or psychiatric disorders. Healthy YCs were under 40 years of age with normal cognitive function (MMSE) and no history of neurological or psychiatric disorders.

All participants underwent clinical interviews, neurological and neuropsychological examinations, and laboratory tests including complete blood count, blood chemistry, thyroid function tests, syphilis serology, and vitamin B12/folate levels. The absence of structural lesions including cerebral infarctions, brain tumors, vascular malformations, and hippocampal sclerosis was confirmed based on brain MRI.

The Institutional Review Board of Samsung Medical Centre (SMC) approved the study protocol, and all methods were performed according to approved guidelines. Written consent was obtained from each participant.

### MRI data acquisition

Standardized three-dimensional (3D) T1turbo field echo images were acquired from all participants at SMC using the same scanner (Achieva 3.0-T MRI 164 scanner, Philips, Best, the Netherlands) and the following parameters: sagittal slice thickness, 1.0 mm with 50% overlap; no gap; repetition time, 9.9 ms; echo time, 4.6 ms; flip angle, 8°, and matrix size, 240 × 240 pixels reconstructed to 480 × 480 over a field of view of 240 mm.

### Aβ PET data acquisition

Participants underwent FBB PET and FMM PET at SMC using a Discovery STe PET/computed tomography (CT) scanner (GE Medical Systems, Milwaukee, WI, USA) in 3D scanning mode that examined 47 slices of 3.3-mm thickness spanning the entire brain^22^. Paired FBB and FMM PET images were acquired on two separate days; mean interval times (4.0 ± 3.4 months across all groups) among the groups were not different (*p* = 0.92). FBB PET was performed first in half of the participants (total 46; 7 ADD, 10 MCI, 16 OCs, 9 YCs, and 4 SVaD) and FMM PET first in the other half (total 61; 22 ADD, 17 MCI, 11 OCs, and 11 YCs). CT images were acquired using a 16-slice helical CT system (140 keV, 80 mA; 3.75-mm section width) for attenuation correction. A 20-min emission PET scan in dynamic mode (consisting of 4 × 5 min frames) was performed 90 min after injection of a mean dose of 311.5 MBq FBB or 185 MBq FMM. 3D PET images were reconstructed in a 128 × 128 × 48 matrix with 2 mm × 2 mm × 3.27 mm voxel size using the ordered-subsets expectation maximization (OSEM) algorithm (FBB iterations = 4 and subset = 20; FMM iterations = 4 and subsets = 20).

### Aβ PET imaging analysis

PET images were co-registered to individual MR images normalized to a T1-weighted MNI-152 template using SPM8 in Matlab 2014b (Mathworks, Natick, MA, USA). After standard space registration, the grey matter was divided into 116 regions using the Automated Anatomical Labeling atlas and white matter^23^. The WC was used as the ROI reference for uptake ratio (which is identical to SUVR) and quantify FBB and FMM retention. Global cerebral cortex amyloid retention ratio was assessed from the volume-weighted average SUVR of 28 bilateral cerebral cortical VOI^22,24^. The cerebral cortical VOI chosen for this study consisted of the following areas: bilateral frontal (superior and middle frontal gyri; medial part of the superior frontal gyrus; opercular part of the inferior frontal gyrus; triangular part of the inferior frontal gyrus; supplementary motor area; orbital part of the superior, middle, and inferior orbital frontal gyri; rectus; and olfactory cortex), posterior cingulate gyri, parietal (superior and inferior parietal, supramarginal and angular gyri, and precuneus), lateral temporal (superior, middle, and inferior temporal gyri, and Heschl’s gyri), and occipital (superior, middle, and inferior occipital gyri; cuneus; calcarine fissure; and lingual, and fusiform gyri).

### Aβ PET positivity based on visual assessment

Three experienced doctors (two nuclear medicine doctors and one neurologist) visually quantified FBB and FMM images. For FBB, tracer uptake was assessed according to the regional cortical tracer uptake system in four brain regions (frontal cortex, posterior cingulate cortex/precuneus, parietal cortex, and lateral temporal cortex). The global uptake in the brain was assessed according to the brain amyloid plaque load system^25^. For FMM, each doctor scored the frontal, temporoparietal/insula, posterior cingulate/precuneus, lateral temporal, and striatum as positive or negative and recorded the overall amyloid status. A scan was categorized as positive if there was uptake in any region. A scan was categorized as negative if there was no uptake in all five regions^13^.

Inter-rater agreement was excellent for FBB (Fleiss k = 0.86) and FMM (Fleiss k = 0.78). After individual ratings were performed, the final visual positivity was determined based on the majority of agreement regarding visual reading results.

### Aβ PET positivity based on SUVR assessment

SUVR positivity was classified based on SUVR cut-off value calculated using the iterative outlier approach in different samples consisting of cognitively normal participants over 55 years of age^26^. To calculate the SUVR cut-off value for Aβ positivity, 171 FMM PET and 202 FBB PET scans were evaluated. Consequently, when WC was used as the reference region, the cortical SUVR cut-off value was 1.1 for FBB and 1.03 for FMM.

For direct comparison of the FBB-FMM conversion method, SUVR values for the FBB-FMM cortical target volume of interest (CTX VOI) were directly converted into CL units using the dcCL method based on the CL conversion equation below^11,27,28^:

$$CL = 100 \times \left( {SUVR_{ind} - SUVR_{YC - 0} } \right)/\left( {SUVR_{ADCI - 100} - SUVR_{YC - 0} } \right)$$

where SUVR_ind_ represents the individual SUVR values of all YC-0 and ADCI-100 participants, and SUVR_YC-0_ and SUVR_ADCI-100_ represent each group’s mean SUVR values. The CL equation was derived for FBB and FMM PET separately and applied to the FBB and FMM SUVR, respectively, from the FBB-FMM CTX VOI. The SUVR from the FBB-FMM CTX VOI used to determine dcCL was termed dcSUVR. When WC was used as the reference region, the dcCL cut-off value was 24.9 dcCL units for FBB and 15.1 dcCL units for FMM.

### Statistical analysis

Analysis of variance (ANOVA) was performed for continuous demographic variables, and the chi-square test was performed for categorical variables. The Fleiss kappa value was calculated for inter-rater reliability. The McNemar test was used to compare the false-positive and false-negative rates between FBB and FMM. MedCalc Statistical Software version 19.1 (MedCalc Software, Ostend, Belgium; https://www.medcalc.org; 2019) was used for the for the chi-square test, ANOVA, and McNemar test and R v3.4.1 (Institute for Statistics and Mathematics, Vienna, Austria; www.R-project.org) was used for Fleiss kappa.

## Data Availability

The data that support the findings of this study are available from the corresponding author upon reasonable request.
