# Author Correction: Concordance in detecting amyloid positivity between ^18^F-florbetaben and ^18^F-flutemetamol amyloid PET using quantitative and qualitative assessments

**DOI:** 10.1038/s41598-021-85177-7

**Published:** 2021-03-03

**Authors:** Soo Hyun Cho, Yeong Sim Choe, Young Ju Kim, Byungju Lee, Hee Jin Kim, Hyemin Jang, Jun Pyo Kim, Young Hee Jung, Soo-Jong Kim, Byeong C. Kim, Gill Farrar, Duk L. Na, Seung Hwan Moon, Sang Won Seo

**Affiliations:** 1 Department of Neurology, Samsung Medical Center, Sungkyunkwan University School of Medicine, Seoul, Republic of Korea; 2 Department of Neurology, Chonnam National University Medical School, Chonnam National University Hospital, Gwangju, Korea; 3 Department of Health Sciences and Technology, SAIHST, Sungkyunkwan University, Seoul, Korea; 4 Neuroscience Center, Samsung Medical Center, Seoul, Korea; 5 Department of Neurology, Yuseong Geriatric Rehabilitation Hospital, Pohang, Korea; 6 Department of Neurology, Myoungji Hospital, Hanyang University, Goyangsi, Korea; 7 Pharmaceutical Diagnostics, GE Healthcare, Chalfont St Giles, UK; 8 Stem Cell and Regenerative Medicine Institute, Samsung Medical Center, Seoul, Korea; 9 Department of Nuclear Medicine, Sungkyunkwan University School of Medicine, Samsung Medical Center, 81 Irwon-ro, Gangnam-gu, Seoul, 06351 Republic of Korea; 10 Samsung Alzheimer Research Center, Samsung Medical Center, Seoul, Korea; 11 Department of Intelligent Precision Healthcare Convergence, Sungkyunkwan University School of Medicine, Suwon, Korea

Correction to: *Scientific Reports* 10.1038/s41598-020-76102-5, published online 11 November 2020

The Acknowledgements section in this Article is incomplete.

“This research was supported by a National Research Foundation of Korea (NRF) Grant funded by the Korean government (MSIT) (No. NRF-2019R1F1A1060353), Brain Research Program through the National Research Foundation of Korea (NRF) funded by the Ministry of Science, ICT & Future Planning (2016M3C7A1913844), grant of the Korean Health Technology R&D Project, Ministry of Health & Welfare, Republic of Korea (HI19C1132), Fourth Stage of Brain Korea 21 Project in Department of Intelligent Precision Healthcare, National Research Council of Science & Technology (NST) grant by the Korea government (MSIP) (No. CRC-15-04-KIST) and Chonnam National University Hospital Biomedical Research Institute (BCRI20012).”

should read:

“This research was supported by a National Research Foundation of Korea (NRF) Grant funded by the Korean government (MSIT) (No. NRF-2019R1F1A1060353/NRF-2019R1A5A2027340), Research of Korea Centers for Disease Control and Prevention (No. 2018-ER6202-02), Brain Research Program through the National Research Foundation of Korea (NRF) funded by the Ministry of Science, ICT & Future Planning (2016M3C7A1913844), grant of the Korean Health Technology R&D Project, Ministry of Health & Welfare, Republic of Korea (HI19C1132), Fourth Stage of Brain Korea 21 Project in Department of Intelligent Precision Healthcare, National Research Council of Science & Technology (NST) grant by the Korea government (MSIP) (No. CRC-15-04-KIST) and Chonnam National University Hospital Biomedical Research Institute (BCRI20012).”

